# What every urologist should know about surgical trials Part II: What are the results and should I apply them to patient care?

**DOI:** 10.4103/0970-1591.44245

**Published:** 2008

**Authors:** Sohail Bajammal, Mohit Bhandari, Philipp Dahm

**Affiliations:** University of Calgary, Department of Surgery, Calgary, Alberta; 1 McMaster University, Department of Surgery, Hamilton, Ontario, Canada; 2 University of Florida, Department of Urology, Gainesville, Florida, USA

**Keywords:** Bibliographic, databases, evidence-based medicine, information storage and retrieval

## Abstract

Surgical interventions have inherent benefits and associated risks. Before implementing a new therapy, we should ascertain the benefits and risks of the therapy, and assure ourselves that the resources consumed in the intervention will not be exorbitant.

**Materials and Methods:**

We suggest a three-step approach to the critical appraisal of a clinical research study that addresses a question of therapy. Readers should ask themselves the three following questions: Are the study results valid, what are the results and can I apply them to the care of an individual patient. This second review article on surgical trials will address the questions of how to interpret the results and whether to apply them to patient care.

**Results:**

Once a study has been determined to be valid, one should determine how effective an intervention is using either relative (i.e. risk ratio, relative risk reduction) or absolute measures (i.e. absolute risk reduction, number-needed to treat) of effect size. The reader should then determine the range within which the true treatment effect lies (95% confidence intervals). Having found the results to be of a magnitude that is clinically relevant, one must then consider if the result can be generalized to one′s own patient, and whether the investigators have provided information about all clinically important outcomes. Then, it is necessary to compare the relative benefits of the intervention with its risks. If one perceives the benefits to outweigh the risks, then the intervention may be of use to one′s patient.

**Conclusion:**

Given the time constraints of a busy urological practice, applying this three-tiered approach to every article will be challenging. However, knowledge of the critical steps to assess the validity, impact and applicability of study results can provide important guidance to clinical decision-making and ultimately result in a more evidence-based practice of urology.

## INTRODUCTION

Part I of this review article addressed the very important issue as to whether the study results were likely to be valid, i.e. represent a likely approximation of the truth using a published randomized trial of abdominal sacrocolpopexy combined with Burch colposuspension with abdominal sacrocolpopexy without colposuspension in terms of their urinary stress incontinence three months after surgery as an example.[1] Women were eligible to participate in this study if they had Stage II to IV pelvic organ prolapse, had no symptoms of urinary stress incontinence and no contraindications to colposuspension. Three months after surgery, 23.8% of the women in the Burch group and 44.1% of the control group met one or more of the criteria for stress incontinence (*P*<0.001). In brief, we found this study to be of high quality when considering key methodological safeguards against bias such as concealed random allocation, stratification, blinding, intention-to-treat analysis and completeness of follow-up. Based on our critical appraisal, we determined the study was likely to yield valid results. The second part of this review article will therefore focus on the equally important topics which are what the results are and how to determine whether these results can and should be applied to the care of an individual patient.

## WHAT ARE THE RESULTS?

### How large was the treatment effect?

Outcomes in randomized trials are either continuous (e.g., blood pressure, duration of hospitalization, or points in a functional outcome measure) or dichotomous (e.g., reoperation, infection, or death). Dichotomous outcomes are used more frequently and represented the primary outcome of the study by Brubaker and colleagues.[1] Dichotomous outcomes are events that are either present or absent in the patient. They are usually presented as the proportion of patients who have such events. Since a randomized trial attempts to answer a clinical question in a controlled environment but with a limited number of study subjects, it is impossible to be absolutely certain that the results found would hold true for all patients, even if they had similar baseline characteristics. Hence, researchers have agreed to use the term point estimate to refer to the treatment effect observed in a trial to emphasize the fact that it is an estimate of the truth. It is important to consider how large and precise this point estimate is. In the following paragraphs, we will discuss the basic statistical methods that the reader can utilize to determine the magnitude of the treatment effect and the precision of such effect in a clinical context.

Although it makes intuitive sense to understand and appreciate the clinical importance of a study that showed, for example, that 20% of the patients in Group 1 died compared to 50% in Group 2, it is of paramount importance to understand certain statistical terms (absolute risk reduction, relative risk, relative risk reduction, odds ratio, and hazard ratio) to help decision-making in more subtle comparisons.

We will use a hypothetical study example to illustrate the uses of these terms. Table 1 summarizes these terms and the corresponding formulas. Consider a trial that compared two surgical interventions in terms of the proportion of patients with stress incontinence one year after surgery. Let us assume that 20 out of 200 patients in the treatment group (Y) and 40 out of 200 patients in the control group (X) developed stress incontinence one year after surgery. A simple way of presenting the data would be as the proportion (or percentage) of patients who had the event of interest in each group. In our hypothetical example, the proportion of patients with stress incontinence one year after surgery in the treatment group is 0.1 (Y= 20/200) and in the control group 0.2 (X= 40/200). More commonly, the event rate is presented as a percentage by multiplying the proportion by 100. Another way of presenting the data is the absolute risk reduction (ARR), or risk difference. The absolute risk reduction is simply the absolute difference between the proportion of patients who had the event of interest in the control group (X) and the proportion of patients who had the event in the treatment group (Y).[2,3] In our hypothetical example, the absolute risk reduction is 0.1 (X-Y = 0.2-0.1).

**Table 1:** Hypothetical study example used for the calculation of absolute risk reduction, relative risk, relative risk reduction, and odds ratio

Group	Outcome of interest		Total
		
	Present	Absent	
Treatment	a	b	a+b
Control	c	d	c+d

Formulas: The event rate in the treatment group = Y = a / (a+b) The event rate in the control group = X = c / (c+d) Absolute risk reduction (ARR) = X-Y Relative risk (RR) = Y/X Relative risk reduction (RRR) = (1 - Y/X) × 100 Odds of the outcome in the treatment group = a/b Odds of the outcome in the control group = c/d Odds ratio (OR) = (a/b) / (c/d) = ad / bc Calculations from the Hypothetical Example: First: You read in the results section of a study where 20 out of 200 patients in the treatment group and 40 out of 200 patients in the control group developed stress incontinence one year after surgery. Second: Draw a 2 × 2 table and replace the letters a, b, c, and d in the table above with numbers from the results you read. In this example, the treatment group total number of patients is 200 (i.e. a+b = 200) and the number of patients with the outcome of interest (stress incontinence) in the treatment group is 20 (i.e., a = 20). You calculate the patients in the treatment group who did not have the outcome of interest (i.e. b) by subtracting 20 from 200. Similarly, for the control group the total number of patients is 200 (i.e. c+d = 200) and the number of patients with stress incontinence is 40 (i.e., c = 40). Third: Solve using formulas aforementioned. The event rate in the treatment group = Y = a / (a+b) = 20 / 200 = 0.1 The event rate in the control group = X = c / (c+d) = 40 / 200 = 0.2 Absolute risk reduction (ARR) = X-Y = 0.2 − 0.1= 0.1 Relative risk (RR) = Y/X = 0.1 / 0.2 = 0.5 Relative risk reduction (RRR) = (1 - Y/X) × 100 = (1−0.5) × 100 = 50% Odds ratio (OR) = (a/b) / (c/d) = ad / bc= (20 × 160) / (180 × 40) = 0.44

Another way of presenting the data would be as relative risk (RR). Relative risk is the ratio of the proportion of patients who had the event in the treatment group (Y) to the proportion of patients who had the event in the control group (X). In our hypothetical example, the relative risk is 0.5 (Y/X = 0.1/0.2). Although the use of relative risk per se makes less sense when communicating with patients and counseling them regarding the treatment options, it is helpful to calculate because it leads to a very clinical apprehensible statistical term, relative risk reduction (RRR). Relative risk reduction is simply the complement of relative risk.[2,3] It is expressed as a percentage and calculated using the equation: RRR = (1− relative risk) × 100. In our hypothetical example, the relative risk reduction is 50% [(1− 0.5) × 100]. In other words, you would tell the patient that the new treatment decreased the risk of stress incontinence by 50% in comparison to patients in the control group. Hazard ratio (HR), on the other hand, is basically the relative risk over a period of time, for example in survival analysis.

A final method of presenting data would be as an odds ratio (OR). This method is preferred by statisticians because of mathematical consideration. However, the concept of odds and odds ratio is very difficult to understand clinically. In addition, there is a risk of overestimating the treatment effect if the odds ratio is interpreted as relative risk. In reports of randomized trials, it is advisable to report the results in terms of absolute or relative risk reduction rather than odds ratios to avoid difficulties in clinical interpretation as well as to avoid the risk of overestimating the treatment effect. Odds ratios are better reserved for case-control studies and logistic regression analyses.[4] Despite these shortcomings, we will explain the odds ratio because of its common use in randomized trial reports. In absolute and relative risk reduction calculation, we are looking at the risk of having an event in the treatment and control group. As we have discussed, the risk is calculated as the proportion of patients who had the event among all patients in the assigned group. The calculation of odds ratio is conceptually different. Rather than dealing with risk, it deals with odds. In the treatment group, we estimate the odds of an event by dividing the number of patients who had the event in that group by the number of patients who did not have the event in the same group. We would do the same for the control group. Finally, to determine the odds ratio of the outcome, we divide the odds in the treatment group by the odds in the control group. Table 1 explains the formula for calculating the odds ratio.

Looking at the results section in the study by Brubaker and colleagues,[1] the results were presented as the percentage in each group with a *P*-value of the comparison. Some comparisons were stated in the section as percentages without numerators or denominators, however, all outcomes of the study for each group with a numerator and denominator were detailed in a table.[1] It is good practice for authors of randomized trials to provide actual numbers to the readers, because having numerators and denominators for each group makes constructing a 2×2 table an easy task. The authors used odds ratio appropriately when they conducted logistic regression analysis to adjust for surgeon and presence or absence of a concomitant paravaginal procedure.

We will calculate the ARR, RR and RRR for the primary outcome of the study (i.e. stress incontinence at three months after the surgery) as a practical example. Looking at the first row of the results table in the article by Brubaker and colleagues,[1] we notice that 35 out of 147 patients in the treatment (Burch) group and 67 patients out of 152 patients in the control group developed stress incontinence. According to our 2×2 table, a=35, b=112, c=67 and d=85. The risk of having stress incontinence after three months in the treatment group (Y) is 35/147= 0.238 and in the control group (X) is 67/152= 0.441. The absolute risk difference is X−Y= 0.441−0.238= 0.203 (or 20.3%). The relative risk of having stress incontinence three months after surgery in the Burch group (Y) compared with the control group (X) is Y/X= 0.54. The relative risk reduction is (1−Y/X) × 100= (1−0.54) × 100= 46%. This means that the addition of Burch colposuspension to abdominal sacrocolpopexy reduced urinary stress incontinence by 46% three months after the surgery compared with abdominal sacrocolpopexy alone.

### How precise was the estimate of the treatment effect?

We indicated earlier that a randomized trial attempts to answer a clinical question by estimating a treatment effect expressed by one of the ways detailed in the previous paragraphs (ARR, RRR, or less favorably OR). However, it is important to determine how precise this estimate is. In other words, what is the plausible range for this estimate? For example, if a study shows that the new treatment reduces the outcome by 50% compared with the control group. Accepting the fact that this is an estimate, we would be more interested in the precision of the estimation. Does this reduction range from 30% to 70% or does it range from 5% to 95%? The more precise the estimate, the more confidence we can put in the results.

There are two ways of determining the precision of results: the *P*-value and the confidence interval. The *P*-value is a crude way of assessing the precision. The *P*-value describes how often an apparent treatment effect estimate will occur in a long run of identical trials if in fact no true effect exists. Let us go back to our hypothetical example. If a new treatment reduces the risk of stress incontinence by 50% compared with the control group and has a *P*-value of 0.04, this means that if we repeated the same study 100 times, there is a possibility of finding such difference of 50% or higher in 4 out of 100 studies, purely due to chance, even if there is no true difference between the two groups. The *P*-value is helpful for investigators to determine the sample size. In other words, it helps to determine how many patients they need to enroll in the study to detect a real difference between the two groups and to minimize the risk of detecting a difference due to chance alone. The *P*-value is arbitrarily set to be 0.05. The *P*-value does not help the clinicians to determine the range within which the treatment effect estimate resides. This is accomplished by the confidence interval (CI). The CI is a set of values within which one can be confident that the true value of the point estimate lies.[2,5] Although the breadth of the CI is chosen arbitrarily, by convention a 95% CI is commonly used. A 95% CI means that if we repeat the study 100 times, we will find the point estimate within the range of the CI 95 times. It makes intuitive sense to state that the more patients enrolled in a study, the more confidence we will put in the study results assuming that its methodology was sound. This is partly because the larger the sample size, the narrower the CI is. A narrow CI means more precision.

Confidence intervals are important for interpreting the results of positive as well as negative studies. In a positive study, a study in which the authors conclude that the treatment is effective, the reader should look at the lower boundary of the CI. If the lower boundary of the CI for the point estimate is still clinically important (i.e. large enough for the surgeon to recommend the treatment to the patient), then we can adopt the new treatment. On the other hand, if the lower boundary of the CI is not clinically important (i.e. the benefit is not large enough for the surgeon to recommend the treatment to the patient), then the study cannot be considered definitive, even if the results are statistically significant (*P*< 0.05). Referring back to our hypothetical example, if the relative risk reduction is 50% of stress incontinence in the treatment group compared to the control group and the 95% confidence interval is 30% to 70%, the lower boundary of the confidence interval is 30% which is still a large treatment effect, hence we would recommend the treatment to our patients. With the same relative risk reduction of 50%, if the 95% CI was 5% to 95%, then there is a possibility that if we implement the new treatment, we might find a relative risk reduction of 5%. One should ask if this improvement is clinically significant enough to warrant the application of the new treatment.

If the study is negative, a study in which the authors concluded that there is no difference between the treatment and control groups (*P*-value > 0.05), one should look at the upper boundary of the CI. If the upper boundary of the CI, if true, shows a result that would be clinically important, then the study does not exclude a significant clinically important effect. The study might not have enrolled enough patients to detect such difference. Hence, it is important to critically appraise negative studies as rigorously as positive studies to avoid dismissing a potentially beneficial treatment. This is clearly relevant to the urological literature, as a study by Breau demonstrated that two-thirds of randomized trials with “negative” findings published in leading urological journals were underpowered to make the claim of no effectiveness.[6] Another hypothetical example will help to clarify this concept. Assume that a study showed no statistically significant difference between the treatment and control groups in terms of stress incontinence at one year. The *P*-value was >0.05. The relative risk reduction was 20% with a 95% CI of -10% to 50%. A relative risk reduction of -10% means that the treatment actually increases the outcome of the study (stress incontinence) by 10% compared with the control group; hence, it is more detrimental than the control group. However, since this is a negative study, we will look at the upper boundary, which is 50%. A 50% relative risk reduction is a large treatment effect that may warrant re-investigating the clinical question with another study with a larger sample size and rigorous methodology hoping to detect a statistically and clinically significant difference.

It is easy to decide on the precision of the treatment effect if the CI is given in the study. However, how should the reader decide on the precision if the CI was not given in the study results? There are three approaches to determine the CI. The first approach is to examine the *P*-value. If the p-value is exactly 0.05, then the lower boundary of the 95% CI for the relative risk reduction has to lie close to zero (i.e., no difference between the two groups). The further the *P*-value from 0.05, the further the lower boundary of the relative risk reduction point estimate would be from zero (i.e. more precise beneficial treatment effect). The second approach is to estimate the 95% CI by calculating it using the standard error of the relative risk reduction. The 95% CI will be the relative risk reduction ± (1.96 × the standard error). The third, and most complex, approach to estimate the 95% CI is to ask a statistician to do the calculation if the standard error of the relative risk reduction was not provided. Alternatively, the interested reader can use one of the available online or downloadable confidence interval calculators.[7–9] However, since these calculators use different formulas, the results may differ slightly.

Looking at the results table in the study by Brubaker and colleagues,[1] we can use any of the online CI calculators to determine the 95% CI for RRR for any outcome. We used the Center of Evidence-Based Medicine′s Stats Calculator to get the CI in this study.[7] The RRR of stress incontinence at three months after the surgery for the treatment group compared with the control group is 46% and the 95% CI is 24.1% to 61.6%. Since this is a positive study, we will look at the lower boundary of the confidence interval, 24.1%. Thus, in the worst case scenario, the treatment group will have 24% reduction of the risk of urine incontinence compared with the control group, which makes us confident in the clinical significance of the results of this study.

We also calculated the RRR and 95% CI for urgency symptoms because this was one of the primary outcomes. The RRR for urgency symptoms at three months after the surgery for the treatment group compared with the control group was 15% and the 95% CI was −15% to 37%. Since this is a negative study, we will look at the upper boundary of the confidence interval, 37%. This is a large treatment effect of clinical significance which suggests that if the investigators had recruited more patients, we might have seen a statistically significant difference for urgency symptoms as well. The lower boundary of the CI, −15%, means that the Burch group might, in the worst case scenario, actually increase the frequency of urgency symptoms by 15% compared to the control group.

## THIRD: CAN I APPLY THE RESULTS TO PATIENT CARE?

### Were the study patients similar to my patient?

Before you apply the results of the study to your patients′ care, you must assess the similarity between your patients and the study′s patients. The best way to assess that is by reviewing the inclusion and exclusion criteria of the study. If your patients would have been eligible to participate in the study, in other words, if they meet all the inclusion criteria and none of the exclusion criteria, then you can confidently apply the results of the study to your patients′ care. However, even if the patient does not exactly meet the inclusion and exclusion criteria, the results may be applicable to your patient. In that case, you should ask yourself whether there is any compelling reason to assume that the results of the study would not apply to your patient. Another important aspect of extrapolating the results to your patients′ care is to be aware that most trials are conducted in a controlled fashion; hence, results are not uniformly effective when applied in real life. Some patients may benefit from the interventions, while others may not. In surgical trials, it is important to ask yourself whether you are capable, in terms of technical skills, of replicating the technique performed in the study. This becomes of paramount importance if the study tests a “new” procedure.

Sometimes you are faced with the situation that your patient characteristics fit into a subgroup of the study where the investigators performed a separate analysis and showed a benefit for that subgroup. It is very important that you examine these findings rigorously because investigators commonly test multiple subgroups looking for any significant effect after the data becomes available. This introduces a risk of finding a significant difference only by chance. There are published guidelines to decide whether differences in subgroups are real.[3] In general, we tend to believe that subgroup analysis is true when: 1) The analyses are limited to few important clinically relevant questions, 2) The analyses were planned before starting the study and the hypothesized magnitude and direction of effects stated beforehand, 3) Important predictors or subgroup variables were incorporated into the design of the trial, such as stratification of randomization by these variables, 4) Sample size was inflated to have enough power to detect differences within subgroups, 5) All subgroups, both positive and negative results, were reported, 6) The magnitude of effect within each group is large and statistically significant, 7) The findings are biologically plausible, and 8) the results are reproducible by other studies.

In the study by Brubaker and colleagues,[1] the authors included women planning sacrocolpopexy for Stage II, III, or IV prolapse if they did not have symptoms of stress incontinence. Potential participants were excluded if they were deemed unlikely to benefit from a Burch colposuspension due to urethral hypermobility. It seems that the patient in our scenario fits the inclusion criteria of the study; hence, we can confidently apply the results to our patient.

### Were all clinically important outcomes considered?

We recommend treatment to patients when it provides an important benefit. It is very important to carefully examine the outcomes of a study and assess how clinically important the outcomes are. What we are interested in is the clinical significance of the results more than the mere statistical significance. A statistically and clinically significant decrease in the rate for the need of a second operation for urinary stress incontinence with a new procedure is more important, for example, than a statistically significant improvement of five points in a given quality of life scale of 100 points, the clinical significance of which is unclear.

In the study by Brubaker and colleagues,[1] the authors assessed stress incontinence and urge symptoms three months after surgery as the primary outcome. The authors explicitly stated how the outcomes were assessed. It is important to realize though that three months is a very short time-frame to assess incontinence outcomes in patients that are expecting a long-term cure. Although one-year results were reported in the results section, these were not part of the primary outcomes which represents a serious limitation of this study.

### Are the likely treatment benefits worth the potential harm and costs?

Before we decide to use the results of surgical trial to guide our clinical practice, it is important to consider the adverse outcomes of both treatment and control groups and to compare the probable benefits of both groups against the potential adverse outcomes. A final decision on using the results of the surgical trial in clinical practice will depend on whether the balance between the benefits and risks in addition to the cost of the treatment is worth the efforts from the surgeon and the patients.

A 30% relative risk reduction of an outcome in the treatment group compared with the control group may sound impressive, yet its impact on the patients and the surgeon′s practice may be minimal. This is the basis of a very important concept, known as the number-needed-to-treat (NNT) concept.[3] The NNT is the inverse of the ARR or risk difference (NNT= 1/ARR). The NNT helps to quantify the tradeoff between benefits and potential harms. Referring to our hypothetical example in Table 1, the ARR is 0.1, hence the NNT is 10 (1/0.1). An NNT of 10 means that you need to treat 10 patients to prevent one adverse outcome (in our hypothetical example, it is stress incontinence). Similar to other point estimates, NNT should be presented with its 95% CI. Otherwise, they can be calculated using the same online calculators mentioned earlier.

In the study by Brubaker and colleagues,[1] there was a significant difference in the duration of surgery (the interval between incision and skin closure), in the Burch and control groups, 170±60 min versus 190±55 min, (*P*=0.002) respectively. There was also a significant difference in the estimated intraoperative blood loss in favor of the sacrocolpopexy alone versus sacrocolpopexy with Burch colposuspension (192±125 ml versus 265±242 ml (*P*<0.001), respectively). Following surgery, the percentage of women who had serious adverse events within three months after surgery was similar in the two groups (14.6% in the Burch group and 14.5% in the control group, *P*=0.79). Of those who had the Burch treatment, 4.5% had serious adverse events that were judged to be plausibly related to treatment and for the control group 3.0% (*P*=0.24). The important points to discuss with the patients when deciding which option to choose are the estimated blood loss and the potential need for blood transfusion as well as the duration of the procedure. The worst case scenario for the sacrocolpopexy with Burch colposuspension procedure is that a woman might lose up to 500 ml of blood while a woman undergoing the sacrocolpopexy procedure alone might only lose up to 67 ml of blood in the best case scenario. The duration of the procedure has an implication on both the patient′s choice as well as the cost of the procedure. Luckily, the duration of the procedure and blood loss were the only two significant differences in favor of the control group, which makes the decision-making somewhat easy. In many surgical trials, one has to weigh improvements in quality of life against some major complications. For those studies, NNT comes in to play to help in the decision-making. We calculated the NNT and its 95% CI for the Brubaker and colleagues study for stress incontinence and urge outcome.[1] The NNT for stress incontinence is 4.9 with a 95% CI of 3 to 10. This means that we need to treat five patients with combined abdominal sacrocolpopexy and Burch colposuspension to prevent one stress incontinence outcome three months after the surgery. The NNT for urgency symptoms was 17.4 with a 95% CI of −20 to 6. This means that we need to treat 17 patients using the Burch technique to prevent one urge outcome three months after the surgery. Since this outcome was not statistically significant between the two groups, the number-needed-to-treat abbreviation (NNT) ranges from 6 to −20 patients. A negative NNT indicates that the treatment has a harmful effect; hence, it is called the number-needed-to-harm (NNH). In this study, using the Burch technique, we need to treat 20 patients to cause harm (urge outcome) in one patient, in the worst case scenario within the CI boundaries.

### Scenario wrap-up

After carefully going over the report of the study by Brubaker and colleagues[1] and following the proposed three-step guideline to critically appraise a randomized controlled trial, we can make an informed judgment about the study. As far as the validity of the results is concerned, we are confident that the investigators implemented all the possible measures to ensure the validity of the results. The only minor concern was the method of allocation concealment, which was not reported. In regards to the second question, what the results of the primary analysis were, the addition of the Burch procedure reduced short-term urinary stress incontinence three months after surgery by 46% compared to abdominal sacrocolpopexy alone, with 95% CI from 24.1% to 61.6%. This translates to an NNT (95% CI) of 5 (3, 10). Finally, regarding our ability to generalize the study findings and applying them to our patient, we found that our patient was similar to the study subjects.

As far as the balance between the benefits and harm goes, we would tell the patient in our scenario upon her return visit that she would most likely benefit from the addition of the Burch procedure by decreasing her risk of stress incontinence by 25% to 60%, at the expense of a slightly longer procedure time, somewhat increased blood loss and a potentially higher rate of urgency symptoms postoperatively. Meanwhile, one caveat of the study was that it did not address long-term outcomes. The fact that we have little information about the expected outcomes beyond three months is very important and is something we should share with our patient when discussing treatment options. We must also realize that this study did not provide us with any information on alternative procedures for stress urinary incontinence other than the Burch procedure, such as minimally-invasive sling procedures, nor did it address costs. This example illustrates that even a high-quality randomized controlled trial with highly valid results, may do little to inform clinical decision-making if important treatment alternatives are not included. Therefore, although the potential benefits of a concomitant Burch procedure appear to outweigh its potential for harm, the patient may or may not choose to undergo a concomitant Burch procedure based on these considerations. This is entirely consistent with an evidence-based practice and relates to the second guiding principle that the best available evidence needs to be integrated with an individual patient′s values and preferences. We should also keep in mind that ideally, clinical decision-making should be based on more than one single study as reviewed in this example, but on the entire body of evidence, ideally a series of related, high-quality studies that have been summarized in a systematic review or meta-analysis.

## CONCLUSIONS

In this two-part article, “What Every Urologist Should Know about Surgical Trials”, we have outlined an approach on how to critically appraise a clinical research study that relates to surgical therapy. The reader should assess the validity of the article, understand the results and determine whether the findings can be applied to their patients. All three aspects of the critical appraisal process are equally important and should therefore be given due consideration. In an evidence-based decision-making process, the urologist should then seek to integrate this information with the specific clinical circumstances and the patient′s individual values and preferences.

## References

[1] Brubaker L, Cundiff GW, Fine P, Nygaard I, Richter HE, Visco AG (2006). Abdominal sacrocolpopexy with burch colposuspension to reduce urinary stress incontinence. N Engl J Med.

[2] Last JM (2001). A dictionary of epidemiology.

[3] Guyatt GH, Rennie D (2002). Users′ guide to the medical literature.

[4] Deeks J (1998). When can odds ratios mislead? Odds ratios should be used only in case-control studies and logistic regression analyses. BMJ.

[5] Montori VM, Kleinbart J, Newman TB, Keitz S, Wyer PC, Moyer V (2004). Tips for learners of evidence-based medicine: 2, Measures of precision (confidence intervals). CMAJ.

[6] Breau RH, Carnat TA, Gaboury I (2006). Inadequate statistical power of negative clinical trials in urological literature. J Urol.

[7] Center for Evidence-Based Medicine Stats calculator. University Health Network.

[8] Center for Evidence-Based Medicine Ebm calculator v1.2. University Health Network.

[9] Herbert R Confidence interval calculator v4.

